# Synthesis of (macro)heterocycles by consecutive/repetitive isocyanide-based multicomponent reactions

**DOI:** 10.3762/bjoc.15.88

**Published:** 2019-04-15

**Authors:** Angélica de Fátima S Barreto, Carlos Kleber Z Andrade

**Affiliations:** 1 Universidade de Brasília, Instituto de Química, Laboratório de Química Metodológica e Orgânica Sintética (LaQMOS), 70910-970 Brasília-DF, Brazil

**Keywords:** consecutive/repetitive multicomponent reactions, isocyanide, isocyanide-based multicomponent reactions, (macro)heterocycles, Passerini reaction, Ugi reaction

## Abstract

Isocyanide-based multicomponent reactions are a versatile tool in the synthesis of heterocycles. This review describes recently developed approaches based on the combination of consecutive or repetitive isocyanide-based multicomponent reactions for the synthesis of structurally diverse heterocycles. These strategies have also allowed the synthesis of a plethora of macroheterocycles in a reduced number of steps.

## Introduction

Isocyanide (isonitrile) chemistry was first described by Lieke in 1859 [1] and forms the basis of a large group of reactions in organic chemistry, especially in isocyanide-based multicomponent reactions (IMCRs) [2–3], such as the Passerini and Ugi reactions, which are reactions that have been widely used in the synthesis of peptides, peptidomimetics and heterocycles [4–8]. In this review, we describe synthetic sequences involving repetitive or consecutive IMCRs that have provided molecules with even more structural diversity. By “repetitive” we mean processes in which two or more IMCRs are occurring in the same reaction vessel using polyfunctionalized compounds. In contrast, consecutive processes involve the use of distinct IMCRs in different stages of a synthetic sequence. This latter strategy requires that the IMCR products have at least one functional group that can be used directly or be manipulated for the subsequent IMCR reaction. These strategies have proved very efficient in the fast obtention of (macro)heterocycles, and the number of examples from the literature has been increasing.

## Review

### Multicomponent reactions (MCRs)

**Scheme 1:**
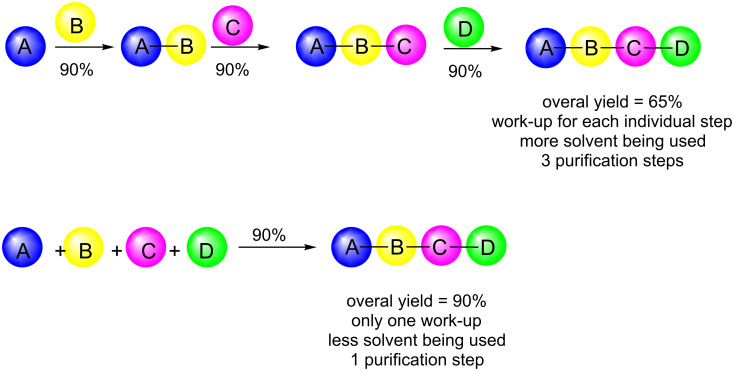
Comparison between a normal sequential reaction and an MCR.

Multicomponent reactions are reactions in which three or more compounds are reacted yielding a product that retains most of the atoms of the starting materials in an atom-economic process [9–11]. A high level of molecular complexity can be generated in a single step and, by varying the structure of each component, different libraries of molecules can be easily obtained. Compared to a sequential synthesis, this strategy presents several other advantages besides atom economy, such as higher overall yields, easiness of procedure and work-up [12], less solvent being used, fewer residues being produced, fewer purification steps and time-saving, contributing to a more sustainable process (Scheme 1).

The Strecker synthesis of α-amino cyanides, reported in 1850, is considered to be the first example of an MCR [13]. Since then, several different MCRs have been reported, including the well documented isocyanide-based MCRs (IMCRs). These particular MCRs take advantage of the unique properties of the isocyanide functional group, which is able to undergo both electrophilic and nucleophilic reactions at the carbon atom. The Ugi reaction, firstly reported by Ugi et al. in 1959 [14], involves an amine, a ketone or aldehyde, an isocyanide, and a carboxylic acid to form a dipeptide product. It is undoubtedly one of the most important IMCRs known and has found many applications in synthetic organic chemistry [2–3,9–10]. Among the IMCRs the Ugi reaction has been the most used in the repetitive/consecutive strategy for the synthesis of (macro)heterocycles. The union of different types of MCRs for the synthesis of more complex products has been reviewed [15–16] and this review will focus only on IMCRs.

### Consecutive IMCRs

#### Synthesis of small-ring heterocycles (tetrazoles, ketopiperazines, imidazoles, imidazolines and thiazoles)

**Scheme 2:**
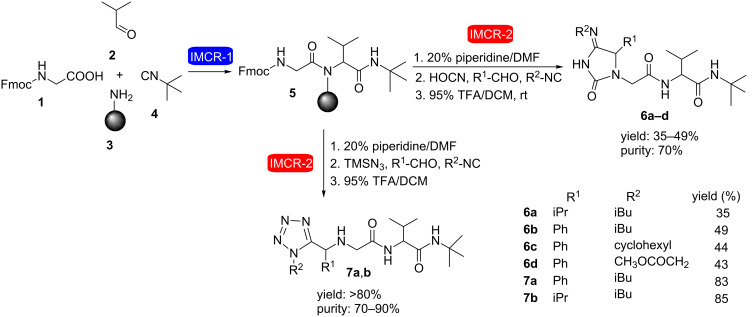
Synthesis of tetrazoles and hydantoinimide derivatives by consecutive Ugi reactions [17].

The use of consecutive Ugi reactions in the synthesis of heterocycles was first described in 2001 by Ugi and Constabel [17] who developed a solid phase strategy to obtain tetrazoles and hydantoinimide derivatives successfully (Scheme 2). In each case, the synthetic sequence began with a classical Ugi reaction between *N*-Fmoc glycine (**1**), isobutyraldehyde (**2**) and *tert*-butyl isocyanide (**4**) in the presence of polystyrene resin **3** as the amine component. After resin removal with piperidine in DMF, a combinatorial strategy of replacing the carboxylic acid component with trimethylsilyl azide (TMSN_3_) (azido-Ugi reaction) or cyanic acid, followed by Fmoc removal (TFA), allowed the formation of the tetrazole (yields >80%) or hydantoinimide nuclei (yields 35–50%), respectively.

**Scheme 3:**
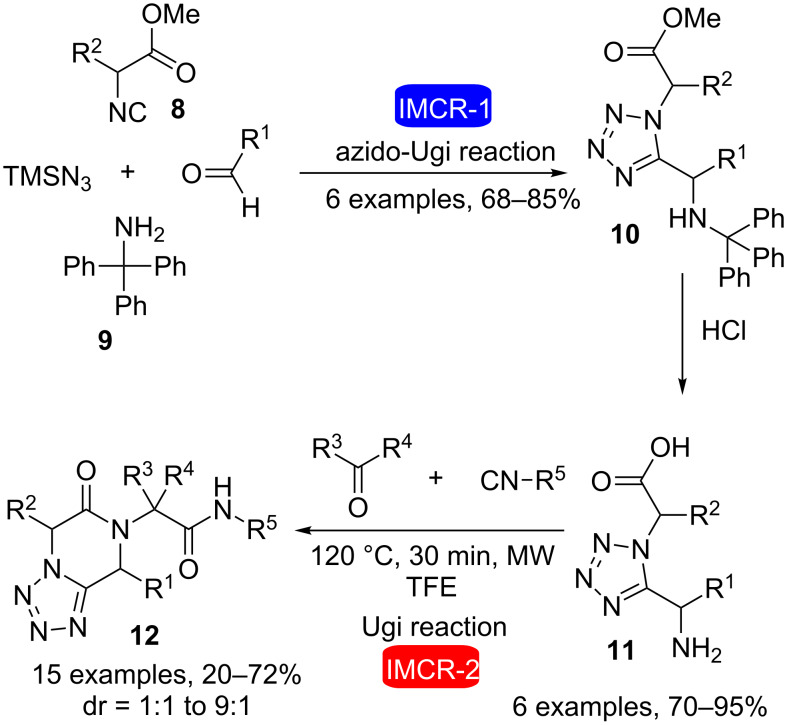
Synthesis of tetrazole-ketopiperazines by two consecutive Ugi reactions [19].

Isocyanoacetate derivatives **8** are efficient building blocks for the synthesis of structurally complex products and biologically active molecules [18]. After an initial IMCR reaction with their isocyanide moieties, hydrolysis of the ester group present in these compounds allows the obtention of carboxylic acids that can be further used in consecutive IMCRs. Furthermore, optically active isocyanoacetates can be easily obtained from natural amino acids. Recently, Dömling et al. [19] used this efficient approach in the synthesis of tetrazole-ketopiperazines (Scheme 3). The strategy involved three steps: first, an Ugi tetrazole reaction between isocyanoacetate derivatives **8**, tritylamine (**9**), various aldehydes and TMSN_3_, followed by treatment of the products with aqueous HCl, which cleaved both the trityl group and the methyl ester, to yield amino acids **11** bearing a 1,5-disubstituted tetrazole. The practicality of the Ugi tetrazole reaction (also called Ugi-azide or azido-Ugi reaction) has been recently reviewed [20–21]. These compounds were then used in an intramolecular three-component four-center Ugi reaction using equimolar amounts of each reagent. Neither room-temperature reactions nor reflux conditions led to satisfactory results. However, microwave heating of **11** at 120 °C for 30 min in trifluoroethanol as solvent allowed the obtention of the desired tetrazole-ketopiperazines **12** in yields ranging from 20–72% (1:1 to 9:1 mixture of diastereomers).

**Scheme 4:**
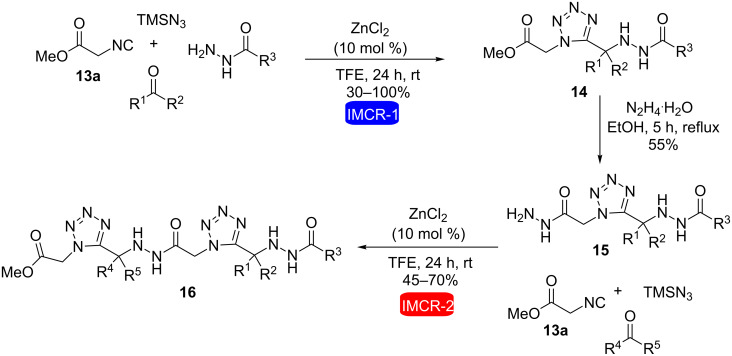
Synthesis of acylhydrazino bis(1,5-disubstituted tetrazoles) through two hydrazine-Ugi-azide reactions and a hydrazinolysis step [22].

Recently, our research group [22] carried out the synthesis of bis(1,5-disubstituted tetrazoles) **14** using two consecutive Ugi reactions (Scheme 4). The synthetic strategy was based on two hydrazino-Ugi-azide reactions and a hydrazinolysis step for the synthesis of acylhydrazino bis(1,5-disubstituted tetrazoles). Methyl isocyanoacetate **13a** was used as an essential component in the first hydrazino-Ugi-azide reaction allowing consecutive Ugi reactions to take place. In the first step, **13a**, hydrazides, aldehyde or ketone, trimethylsilyl azide (TMSN_3_) and ZnCl_2_ (10 mol %) in trifluoroethanol (TFE) were stirred at room temperature for 24 h to obtain acylhydrazino 1,5-disubstituted tetrazoles **14** in 30–100% yield. Attempts of this reaction without the use of catalyst provided the desired products in low yields. Subsequently, a hydrazinolysis reaction with hydrazine monohydrate led to the corresponding hydrazides **15**, which were used in a second hydrazine-Ugi-azide reaction with various ketones, to obtain the acylhydrazino bis(1,5-disubstituted tetrazoles) **16** in yields ranging from 45–70%.

**Scheme 5:**
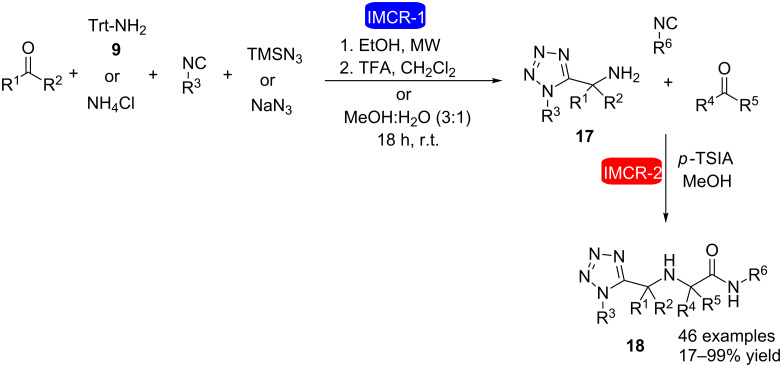
Synthesis of substituted α-aminomethyltetrazoles through two consecutive Ugi reactions (U-4CR and U-3CR) [23].

Consecutive Ugi reactions for the synthesis of substituted α-aminomethyl tetrazoles have also been described (Scheme 5) [23]. The synthetic strategy was based on a four-component Ugi reaction (U-4CR) followed by a three-component Ugi reaction (U-3CR). The first step involved the reaction of ammonium chloride or tritylamine, with oxo components, isocyanide, and sodium azide or TMS azide followed by acid treatment with TFA to obtain α-aminomethyl tetrazoles **17**. Subsequently, a new three-component Ugi reaction was performed involving different amino methyl tetrazoles with different oxo components, isocyanides and *p*-toluenesulfinic acid (*p*-TSIA) in (semi)stoichiometric amounts to obtain substituted α-aminomethyltetrazoles **18** in up to 99% yield.

**Scheme 6:**
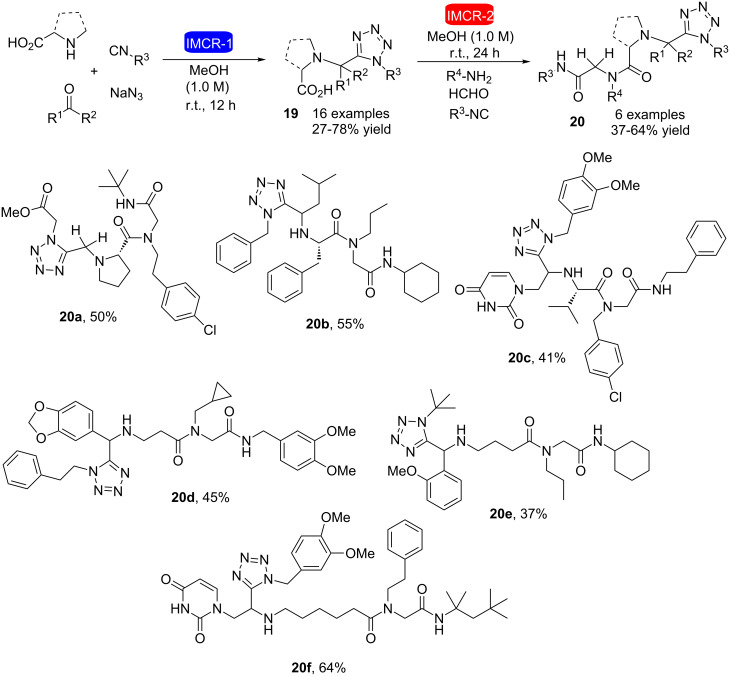
Synthesis of tetrazole peptidomimetics by direct use of amino acids in two consecutive Ugi reactions [24].

Another recent study carried out the synthesis of tetrazole peptidomimetics by the direct use of unprotected amino acids in two consecutive Ugi-type reactions [24]. Acid-tetrazole compounds **19** were obtained using *C*,*N*-unprotected amino acids in an Ugi-tetrazole reaction with oxo components, isocyanide, and sodium azide (Scheme 6). The success of the reaction was evidenced by the sole obtention of the Ugi-tetrazole product without any trace of other Ugi-type reaction products. Sequentially, acid **19** was subjected to a second Ugi reaction with oxo components, amines and isocyanides to obtain six tetrazole peptidomimetics (**20a**–**f**). Highly complex molecules were easily obtained in only two steps (sequential Ugi-tetrazole/Ugi-reaction) without the need for amino acid protection/deprotection reactions.

**Scheme 7:**
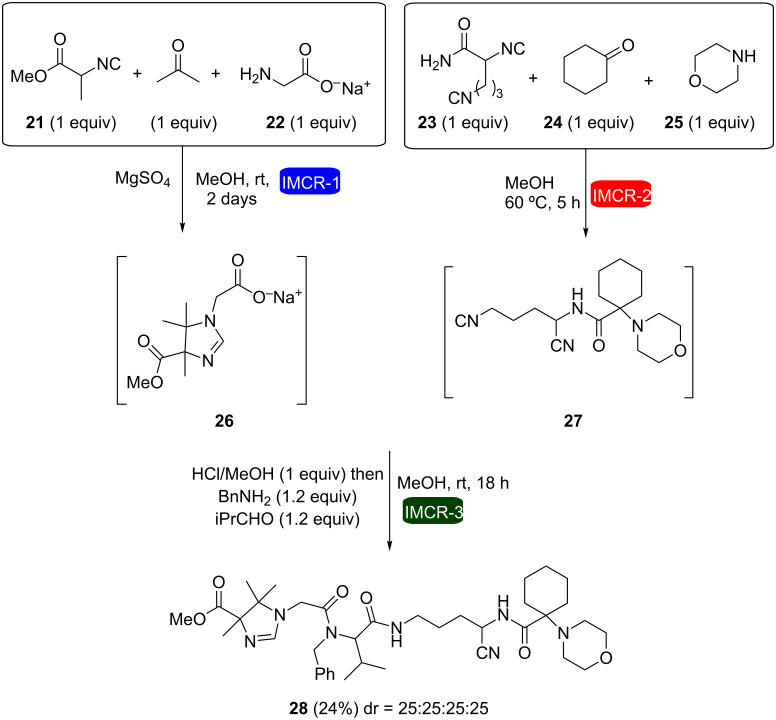
One-pot 8CR based on 3 sequential IMCRs [25].

**Scheme 8:**
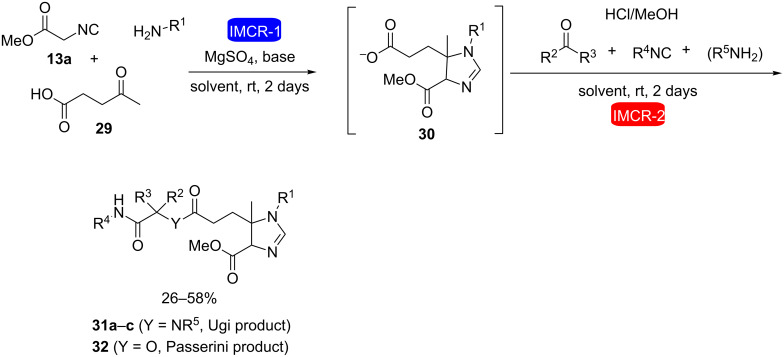
Combination of IMCRs for the synthesis of substituted 2*H*-imidazolines [25].

A remarkable result was described by Orru and co-workers who used two consecutive IMCRs in the same reaction pot to obtain the first example of an eight-component reaction (8CR) [25]. This was accomplished after a careful study of functional-group and solvent compatibilities from previous works of the same group and led to the formation of complex molecules possessing many points of diversity. In this strategy, intermediates **26** and **27** were formed through Ugi-type reactions and then mixed together with benzylamine and isobutyraldehyde to furnish the final product in 24% yield as a mixture of diastereomers (Scheme 7). Other examples of 5- and 6CRs involving Passerini and Ugi reactions such as those represented in Scheme 8 were also reported in the same work.

**Scheme 9:**
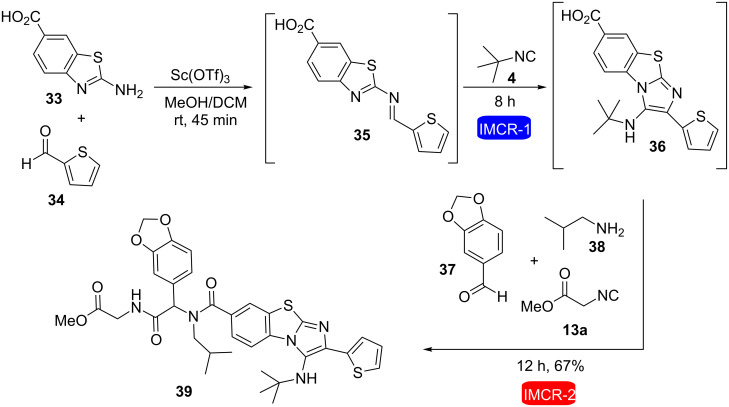
6CR involving a tandem combination of Groebke–Blackburn–Bienaymé and Ugi reaction for the synthesis of a complex heterocycle [26].

**Scheme 10:**
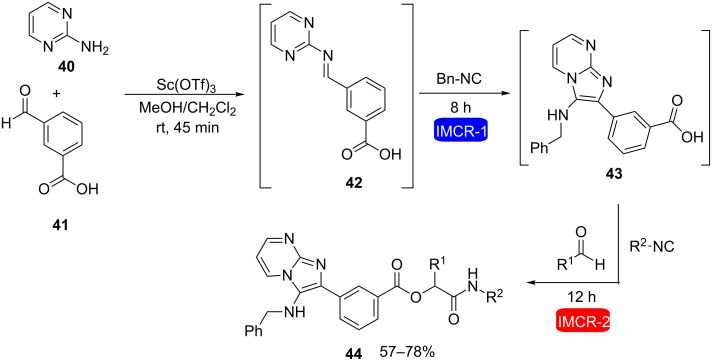
5CR involving a tandem combination of Groebke–Blackburn–Bienaymé and Passerini reaction for the synthesis of a complex heterocycle [26].

Al-Tel et al. described the tandem combination of Groebke–Blackburn–Bienaymé and Ugi or Passerini reactions in the same reaction flask without isolating any intermediate, allowing the preparation of complex heterocycles through sequential additions of five or six components [26]. For instance, compounds **39** and **44** were efficiently obtained in good yields using this strategy (Scheme 9 and Scheme 10, respectively) and many other examples were described in this work.

**Scheme 11:**
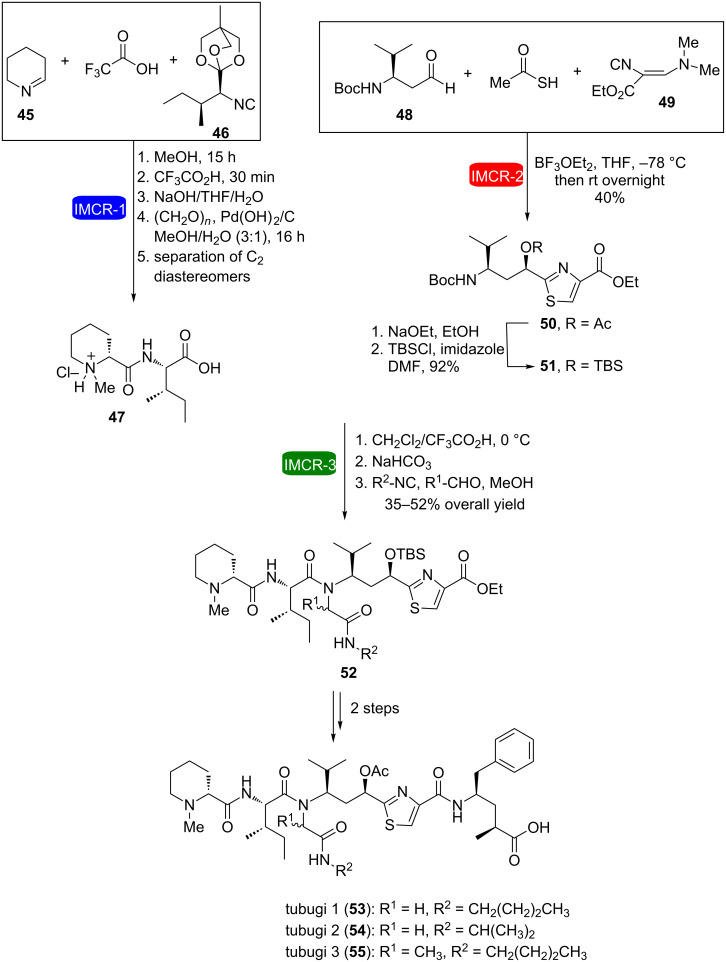
Synthesis of tubugis via three consecutive IMCRs [27].

The strategy of consecutive IMCRs has also been successfully used in the synthesis of tubulysin analogues called tubugis (**53**–**55**) [27]. These molecules are *N*-substituted peptides, which possess a very high cytotoxic activity (on the picomolar range). They were prepared using three different IMCRs (Scheme 11): the Mep-Ileu-OH dipeptide fragment **47** was obtained as a diastereomeric mixture via an Ugi–Nenajdenko reaction using the 4-methyl-2,6,7-trioxabicyclo[2,2,2]octyl (OBO) ester **46** to avoid epimerization of the isocyanide, followed by a reductive amination and chromatographic separation of the isomers; a Passerini–Dömling IMCR led to the heterocyclic fragment of the molecule, called tubuvaline (**50**); and finally an Ugi reaction was used to couple them. Initial attempts to use tubuvaline **50** led to an undesirable product due to water attack at the reaction intermediate before Mumm rearrangement. This was circumvented by changing the protecting group of **50** from acetyl to *tert*-butyldimethylsilyl (TBS, compound **51**). The coupling of the fragments via Ugi reaction was carried out under very controlled conditions, using a syringe pump to slowly add the isocyanide, thus avoiding the formation of the double-addition product.

**Scheme 12:**
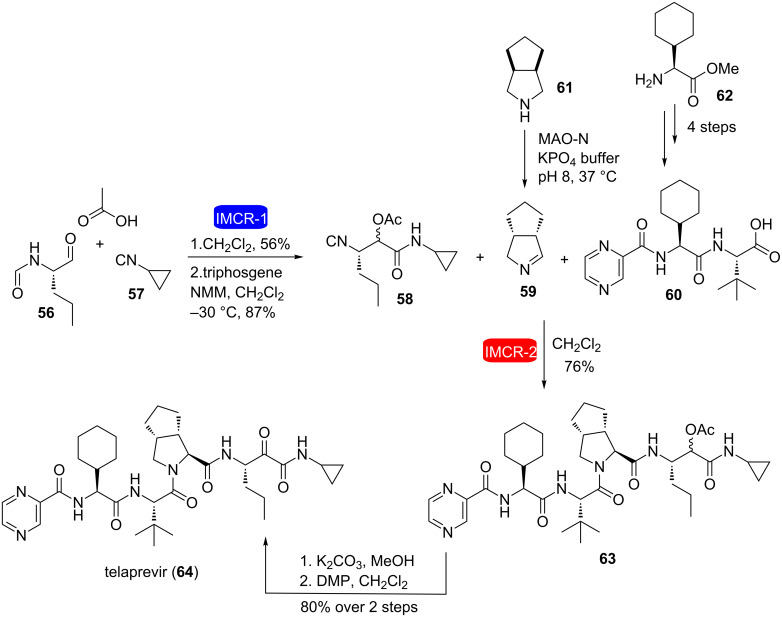
Synthesis of telaprevir through consecutive IMCRs [28].

Ruijter et al. performed the synthesis of telaprevir **64**, a protease inhibitor used in the treatment of hepatitis C, through a very short and efficient synthetic strategy involving as key steps two IMCRs (Ugi and Passerini) [28]. The strategy involved the synthesis of isocyanide **58** via a Passerini reaction using aldehyde **56**, cyclopropyl isocyanide **57** and acetic acid, followed by reaction of the resulting formamide with triphosgene (Scheme 12). Compound **58** was obtained as a 78:22 diastereomeric ratio without any racemization of the pre-existing stereocenter and then used in the key step Ugi-type 3CR with cyclic imine **59** (generated in situ from catalytic oxidation of amine **61**) and pyrazinecarboxylic acid **60** (readily available in four steps from L-cyclohexylglycine methyl ester (**62**)) to give compound **63**, which was converted to telaprevir (**64**) after two additional steps. This approach reduced by more than half the number of steps compared to the existing linear synthetic sequence for telaprevir.

**Scheme 13:**
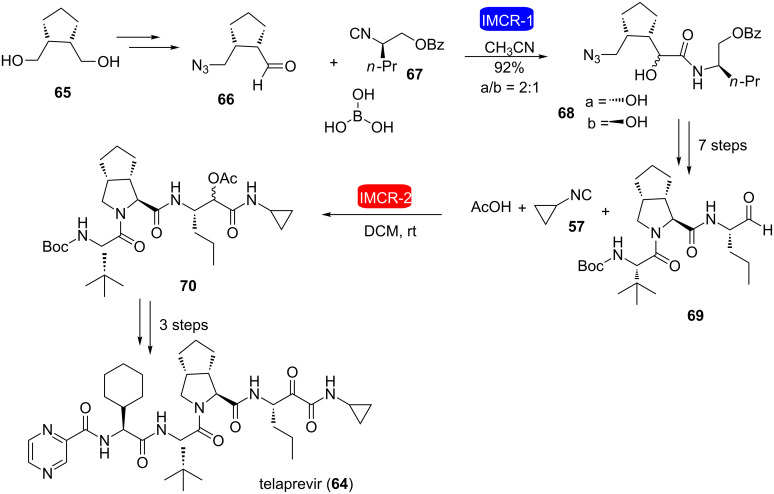
Another synthesis of telaprevir through consecutive IMCRs [29].

Later on, Riva et al. reported an alternative synthesis of telaprevir using two consecutive IMCRs (Scheme 13) [29]. The first one is a Passerini-type reaction between aldehyde **66**, isocyanide **67** and boric acid, which yielded a 2:1 mixture of diastereomers. After converting **68** into aldehyde **69**, this was reacted with cyclopropyl isocyanide **57** and acetic acid (Passerini reaction) furnishing compound **70**, which was converted to telaprevir (**64**) in three additional steps.

#### Macrocyclic peptoid synthesis

**Scheme 14:**
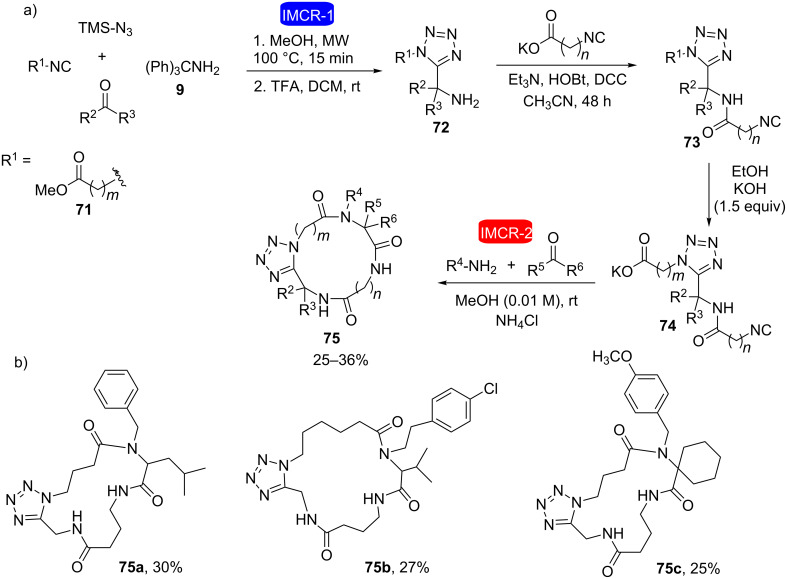
a) Synthetic sequence for accessing diverse macrocycles containing the tetrazole nucleus by the union of two IMCRs. b) Some compounds obtained by this strategy [30].

Dömling et al. performed the combination of two isocyanide-based multicomponent reactions in the synthesis of macrocycles containing a tetrazole nucleus [30]. The strategy was based on the use of α-isocyano-ω-carboxylic esters **71** via a microwave-mediated Ugi-azide reaction at the beginning of the synthesis and an intramolecular Ugi reaction of bifunctional compounds **74** at the end, allowing the obtention of the 16- to 20-membered tetrazolic macrocycles **75** in only five steps (Scheme 14).

**Scheme 15:**
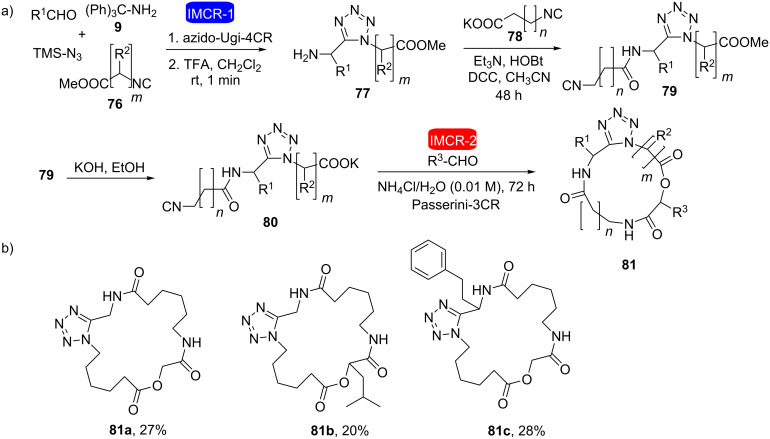
a) Synthetic sequence for the tetrazolic macrocyclic depsipeptides using a combination of two IMCRs (Ugi and Passerini reactions). b) Compounds **81a–c** are representative of the 21 depsipeptides macrocycles obtained [31].

A similar approach was used shortly afterwards by the same research group in the synthesis of macrocyclic depsipeptides containing a tetrazole nucleus [31]. The combination of two isocyanide-based multicomponent reactions (azido-Ugi and Passerini reactions) allowed easy access to a library of macrocyclic depsipeptides in only four steps with variations in the size of the macrocycle as well as in the side chains (Scheme 15). This was the first example in which the intramolecular Passerini reaction was performed using bifunctional isocyanocarboxylate (Scheme 15a). Scheme 15b shows three of the 21 depsipeptides macrocycles synthesized by the authors in this study.

**Scheme 16:**
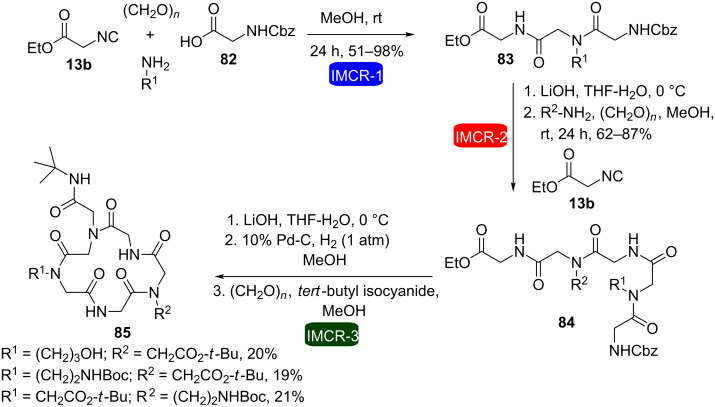
Synthesis of cyclic pentapeptoids by consecutive Ugi reactions [32].

**Scheme 17:**
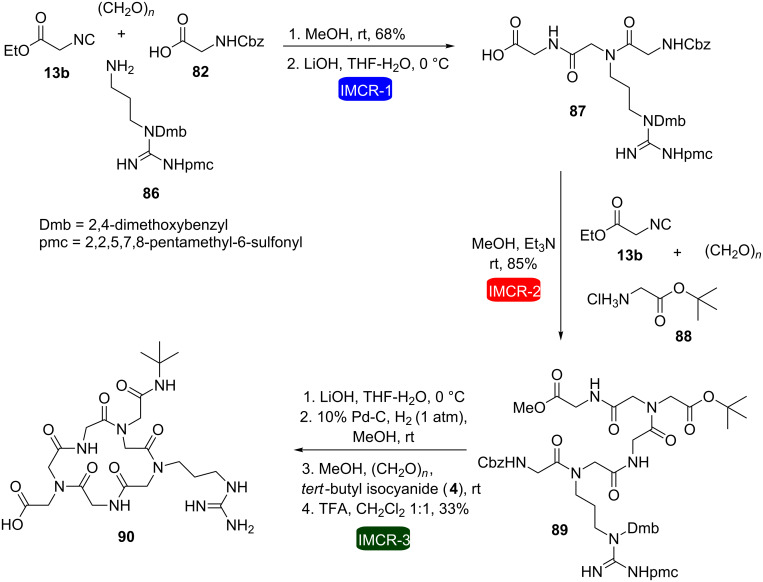
Synthesis of a cyclic pentapeptoid by consecutive Ugi reactions [32].

Ethyl isocyanoacetate (**13b**) has also been successfully used as starting material to allow consecutive Ugi reactions in the synthesis of macrocycles (which were considered as macroheterocycles in the context of this review). In this respect, Wessjohann et al. developed a methodology for the synthesis of cyclic RGD pentapeptoids (RGD = arginine-glycine-aspartic acid) by consecutive Ugi reactions [32]. This was the first example in which the Ugi reactions were used in the construction and cyclization of peptoids in a combined fashion. The methodology was based on two consecutive four-component Ugi reactions for the construction of the acyclic precursors **84** and **89**, followed by a final intramolecular Ugi reaction under pseudo-high dilution conditions (to avoid oligomerization) to furnish cyclopeptoids **85** and **90**, respectively (Scheme 16 and Scheme 17). Ester hydrolysis and *N*-deprotection reactions were carried out in between. In this approach, by varying the amine component, the side chains of the peptoid backbone could be easily exchanged.

**Scheme 18:**
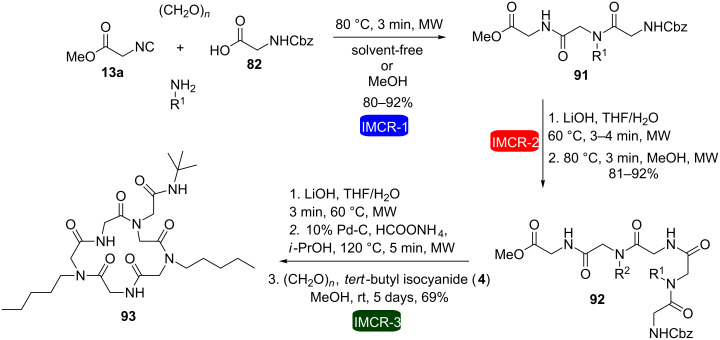
MW-mediated synthesis of a cyclopeptoid by consecutive Ugi reactions [33].

Later on, our group introduced the use of microwave heating to this same synthetic strategy for the synthesis of a cyclic peptoid (Scheme 18) [33]. The combination of these two tools (microwave heating and consecutive IMCRs) has proved to be a particularly attractive method for the synthesis of macroheterocycles, which could be easily accomplished in a reduced number of steps and very short reaction periods (except for the last step). Three consecutive Ugi reactions were performed followed by the respective hydrolysis and deprotection, furnishing an amino acid, which was cyclized in good yield to macrocycle **93** via an intramolecular Ugi three-component four-center reaction (U-3C-4CR).

**Scheme 19:**
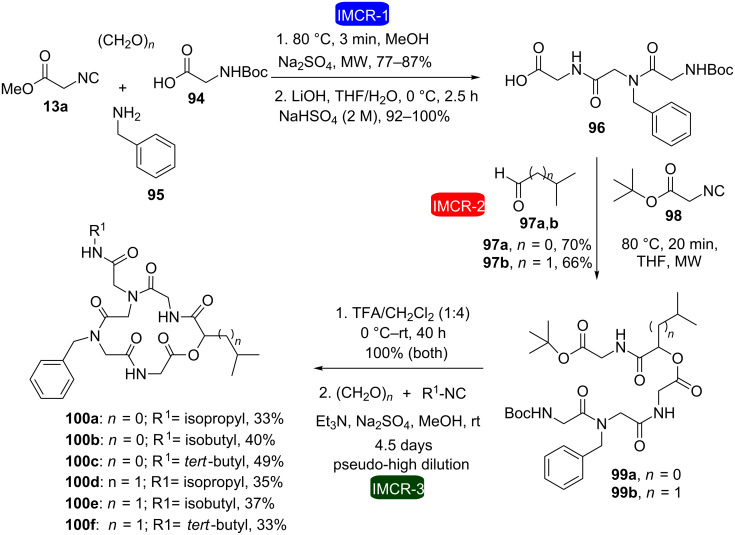
Synthesis of six cyclic pentadepsipeptoids via consecutive isocyanide-based IMCRs [34].

In the continuation of our studies, we used a similar strategy for the combination of consecutive isocyanide-based multicomponent reactions (Ugi and Passerini reactions) [34]. This methodology was used in the synthesis of six cyclic depsipeptoids inspired by the structure of the natural depsipeptide sansalvamide A, which involved five steps (Scheme 19). In the first step, formation of the peptoid was achieved via the first Ugi reaction. Then, subsequent hydrolysis of the ester was followed by formation of an acyclic depsipeptoid via Passerini reaction between acid **96**, isocyanide **98** and aldehydes **97**. Trifluoroacetic acid (TFA) allowed deprotection of the amine/acid groups. In the last step, a macrocyclization reaction via an intramolecular Ugi reaction provided the achievement of the cyclic depsipeptoids **100a–f** in yields ranging from 33–49% depending on the substrate.

**Scheme 20:**
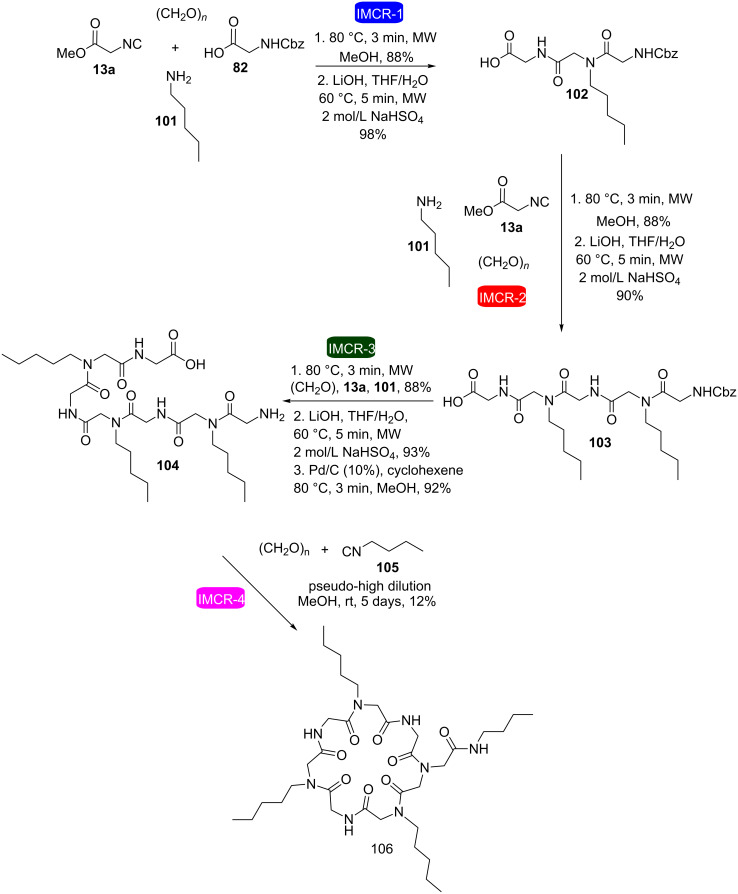
Microwave-mediated synthesis of a cyclic heptapeptoid through four consecutive IMCRs [35].

More recently, our research group described a fast and efficient strategy for the synthesis of macrocycles using four consecutive Ugi reactions (Scheme 20) [35]. This was the first example in the literature in which four consecutive IMCRs were employed. The strategy allowed the synthesis of a cyclic heptapeptoid in only 8 steps using microwave irradiation in seven of these steps allowing short reaction times (3–5 minutes) and excellent yields of the intermediates (88–98%). The non-optimized low yield of the last step was attributed to difficulties during the purification step along with to some oligomerization that might have occurred.

### Repetitive IMCRs – multicomponent macrocyclizations through bifunctional building blocks

**Scheme 21:**
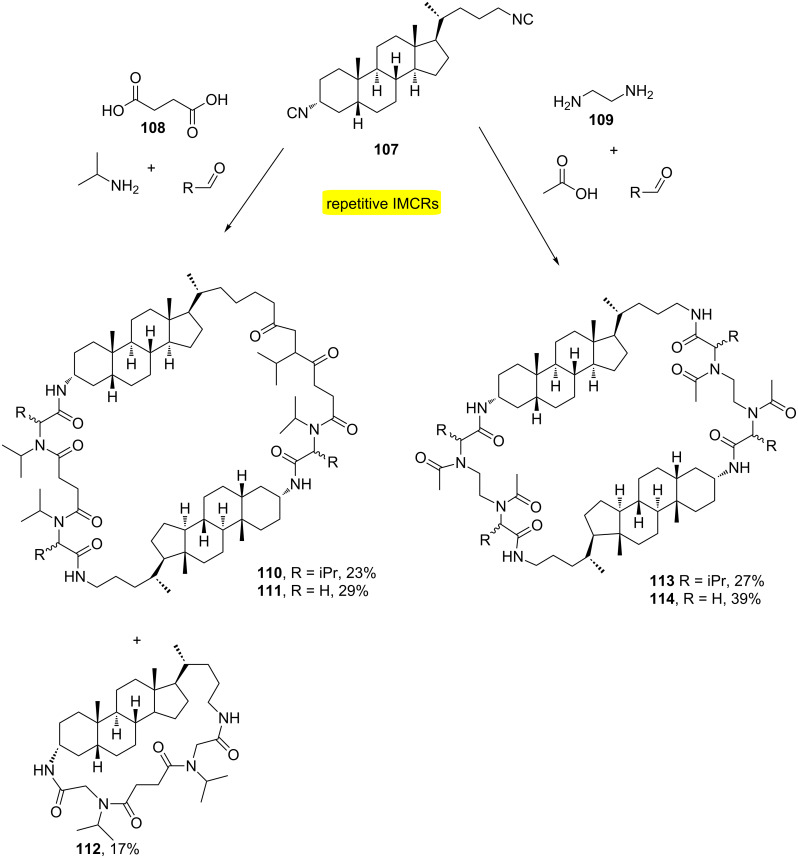
Macrocyclization of bifunctional building blocks containing diacid/diisonitrile and diamine/diisonitrile [37].

A strategy that has been widely used for the synthesis of macroheterocycles is the multiple multicomponent macrocyclizations including bifunctional building blocks (M^3^iB^3^s or MiBs). In this type of strategy, several subtypes of such reactions are conceivable varying the number and the type of IMCRs [36]. In this sense, Wessjohann and co-workers presented a direct method to generate chimeric peptoid macrocycles containing steroid moieties [37]. The process was based on designing a 4-fold Ugi-4CR macrocyclization by using the steroidal diisocyanide **107** with diacid **108** or diamine **109** (Scheme 21). According to the authors, this was the first report of repetitive multicomponent reactions to be used directly to obtain macrocycles of this complexity and size.

**Scheme 22:**
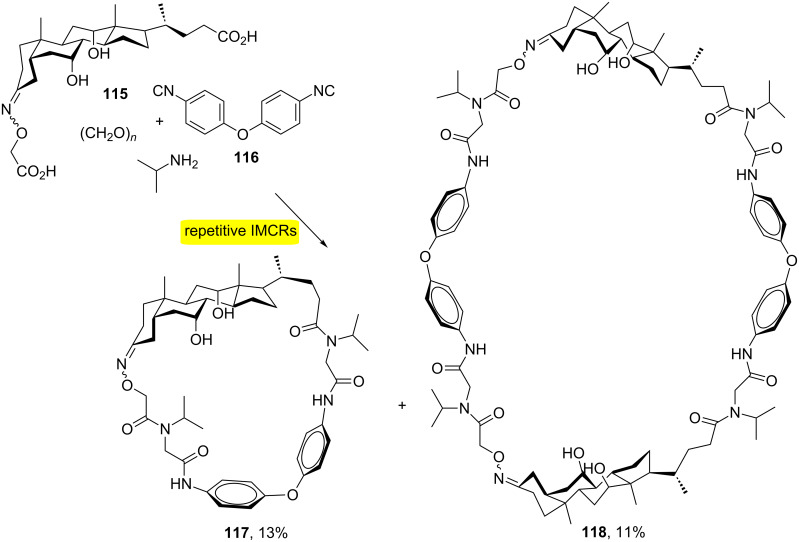
Synthesis of steroid-biaryl ether hybrid macrocycles by MiBs [38].

The synthesis of macrocycles with up to 16 new bonds being formed simultaneously has been described (Scheme 22). The strategy was based on combining steroidal dicarboxylic acids **115** and biaryl ether diisocyanide **116** [38]. The approach provided the synthesis of steroid-biaryl ether hybrid macrocycles **117** and **118** with up to 68 members by the MiBs strategy.

**Scheme 23:**
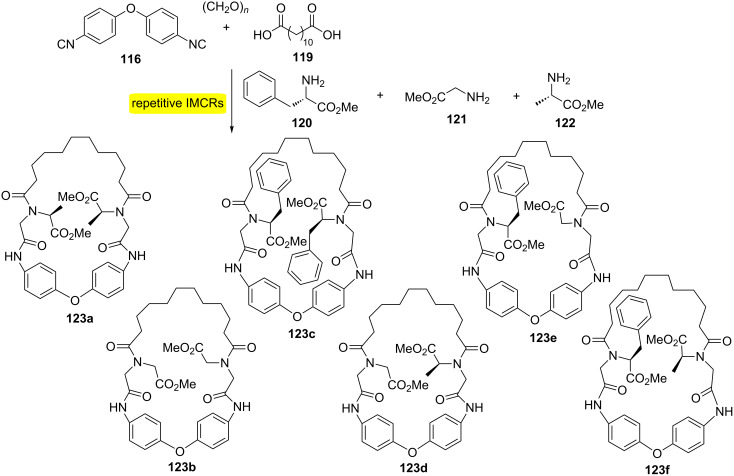
Synthesis of biaryl ether-containing macrocycles by MiBs [39].

Another representative example of MiBs is the synthesis of the biaryl ether-containing macrocycles **123a–f** (Scheme 23) [39]. The synthetic strategy involved the mixing of three different C-protected amino acids (**120**–**122**) with diisocyanide **116** and diacid **119**. The approach allowed the one pot obtention of six different macrocycles and a macrocycle core system containing two symmetrical building blocks.

**Scheme 24:**
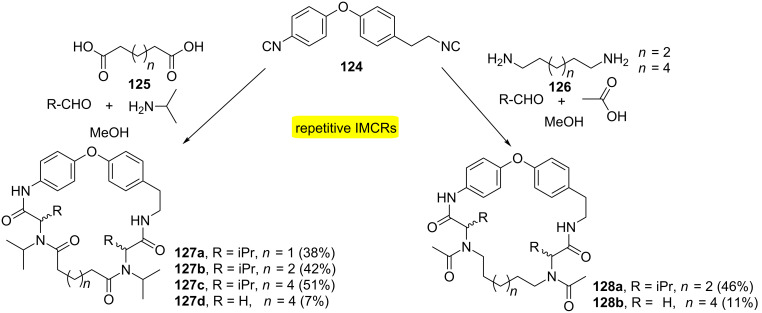
Synthesis of natural product-inspired biaryl ether-cyclopeptoid macrocycles [40].

This same strategy has also been carried out for the synthesis of natural products containing biaryl ether-cyclopeptoid macrocycles **127** and **128** (Scheme 24) [40]. The approach used involved diisocyanides **124** (representing a biaryl ether moiety) reacting with aliphatic diacid/diamine and amine/aldehyde, respectively, to generate the target macroheterocycles.

**Scheme 25:**
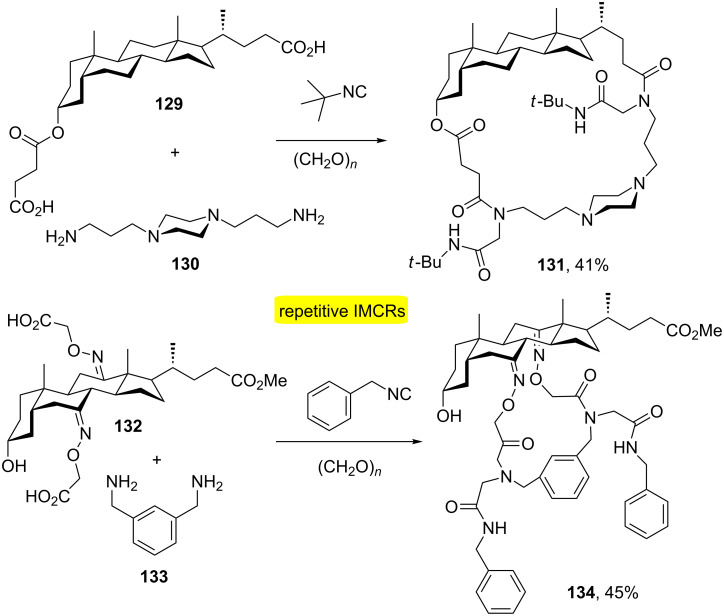
Synthesis of cholane-based hybrid macrolactams by MiBs [41].

Wessjohann and Rivera [41] performed the first use of the diamine/diacid combination of bidirectional Ugi-MiBs in the synthesis of novel steroid–peptoid hybrid macrocycles. Scheme 25 shows two examples using this combination for the synthesis of cholane–peptoid hybrid macrocycles.

**Scheme 26:**
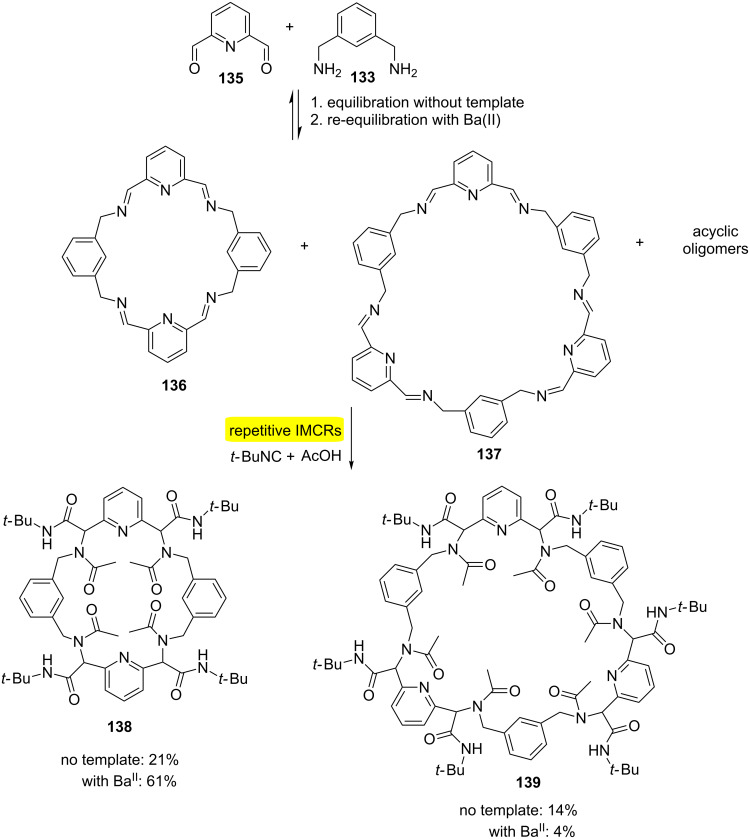
Synthesis of macrocyclic oligoimine-based DCL using the Ugi-4CR-based quenching approach [42].

Another application of the MiBs approach has been found in the use of dynamic combinatorial chemistry (DCC) [42]. One of the most accessible reversible bonds is the imine bond and has been widely used in DCC. In this context, a freezing process of a dynamic combinatorial library (DCL), which is a system of recognition and thermodynamic control, has been developed using multiple Ugi-4CRs (Scheme 26). This approach allows the obtention of macrocycles without the use of pseudo-high-dilution protocol and addition of Ba(II) allowed better results of the final product. This combination has allowed the first selective formation of a 6-fold Ugi-MiB.

**Scheme 27:**
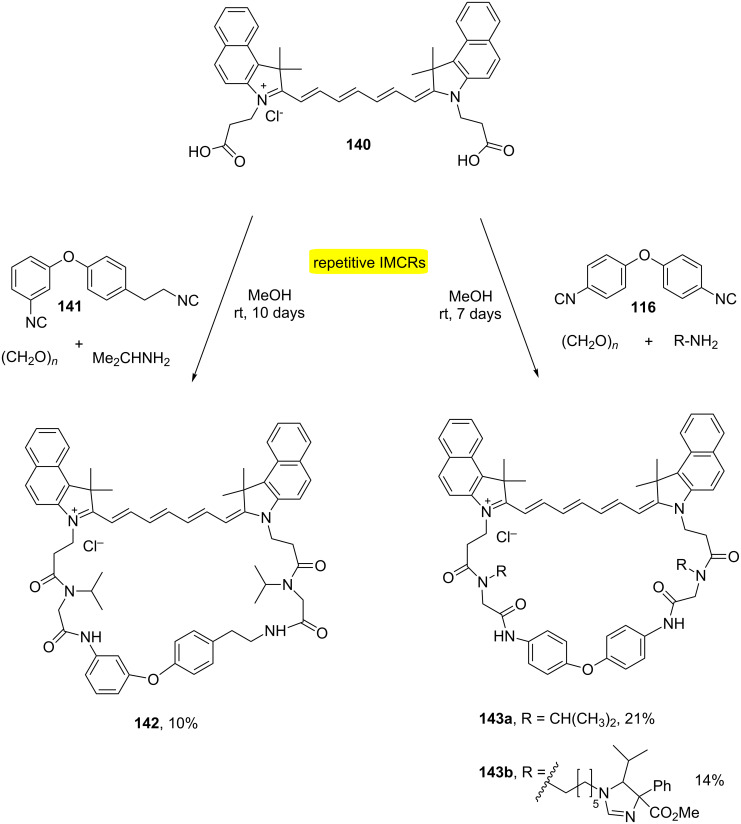
Dye-modified and photoswitchable macrocycles by MiBs [43].

Functional macrocycles have been developed with dye-modified and photoswitchable moieties by MiBs [43]. The approach employed the use of bifunctionalized near-infrared (NIR) dyes containing two carboxylic acid moieties with diisocyanide building blocks providing the formation of somewhat flexible 34- and 35-membered macroheterocycles **142** and **143** in yields ranging from 10 to 21% after long reactional times (Scheme 27).

**Scheme 28:**
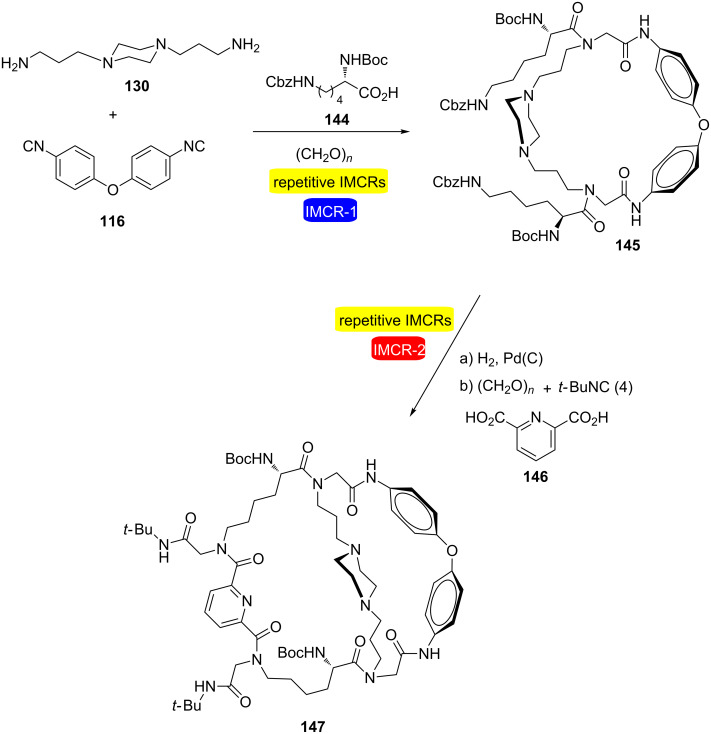
Synthesis of nonsymmetric cryptands by two sequential double Ugi-4CR-based macrocyclizations [44].

There are some interesting examples in which repetitive and consecutive IMCRs were used in a combined fashion. For instance, Wessjohann and Rivera [44] performed the synthesis of nonsymmetric cryptands by two sequential double Ugi-4CR-based macrocyclizations (Scheme 28). The approach also relies on MiBs strategy [45]. The main focus was on the use of the Ugi four-component reaction (Ugi-4CR) due to the tremendous capability of this process to generate molecular complexity. In the approach, one of the building blocks taking part in the first Ugi-MiB must contain a protected, Ugi-reactive functional group to be subsequently activated for the next macrocyclization. In this way, after the first double Ugi reaction between diamine **130**, diisocyanide **116**, paraformaldehyde and acid **144**, Cbz removal of the macrocycle **145** formed led to another diamine intermediate that was involved in a second double Ugi reaction with paraformaldehyde, *tert*-butyl isocyanide and diacid **146**, to yield cryptand **147** in 31% overall yield from **130**. Other macroheterocycles were synthesized in this study using this same protocol.

**Scheme 29:**
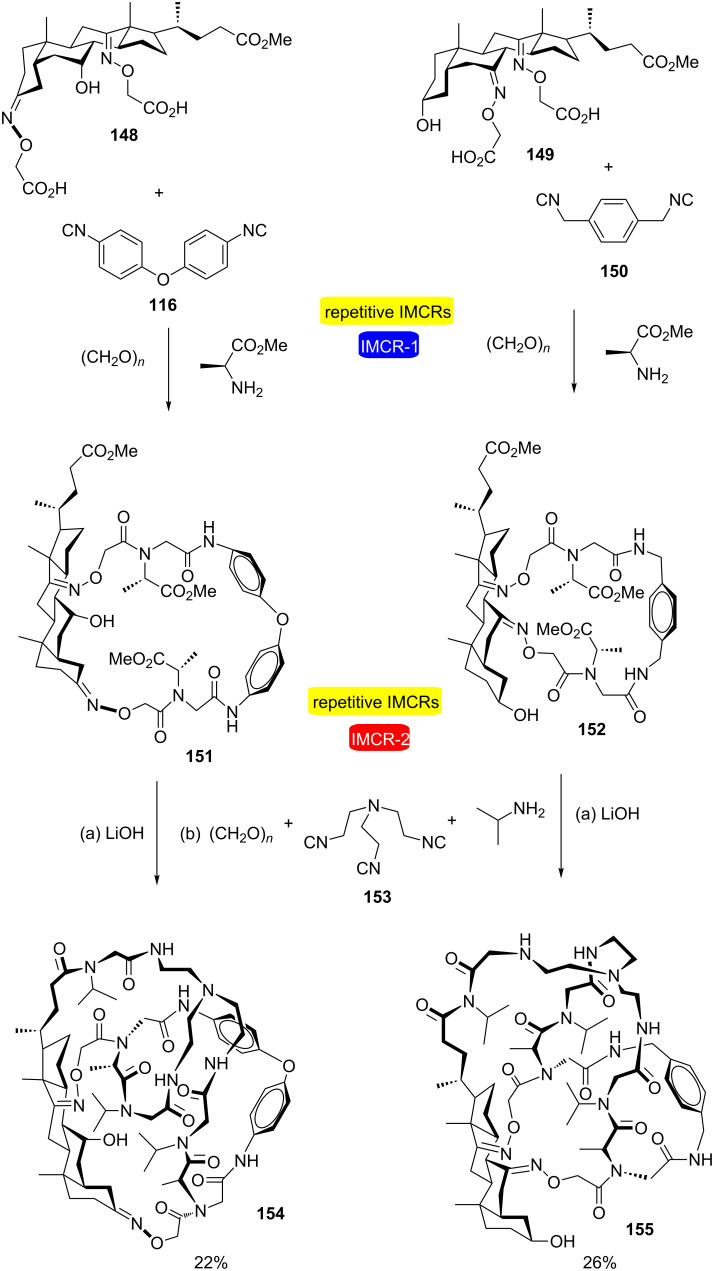
Synthesis of steroid–aryl hybrid cages by sequential 2- and 3-fold Ugi-4CR-based macrocyclizations [46].

Using this same combined approach, Wessjohann and Rivera developed a very efficient strategy for the synthesis of supramolecular compounds via Ugi-type multiple multicomponent macrocyclizations of polyfunctional building blocks (Scheme 29) [46]. An initial double Ugi-4CR-based macrocyclization yielded steroid-aryl hybrid macroheterocycles **151** and **152**, which after ester hydrolysis, acted as trifunctional building blocks for consecutive 3-fold Ugi-4CR-based macrocyclizations with triisocyanide **153**. The cross-linked igloo-shaped skeletons of cages **154** and **155** were obtained in a remarkable one-pot reaction sequence, which involved the incorporation of 13 building blocks and the formation of 20 new bonds without the need of isolating any intermediate in the process.

**Scheme 30:**
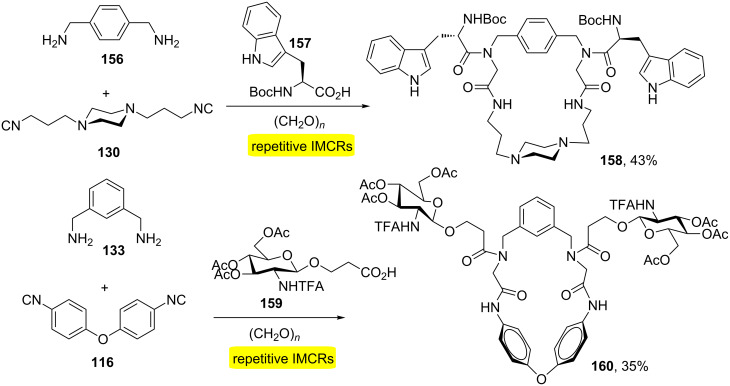
Ugi-MiBs approach towards natural product-like macrocycles [47].

Wessjohann and co-workers demonstrated that the MiBs strategy could be easily used to obtain several bidirectional macrocycles with exocyclic substituents with side chains derived from natural products [47]. The combination of acid components with side chains derived from natural products containing amino acid residues (e.g., Arg, Cys, His, Trp) and sugar, bifunctional components (diamino/diisocyanide) and formaldehyde allowed the rapid production of eight functionalized macrocycles (Scheme 30).

**Scheme 31:**
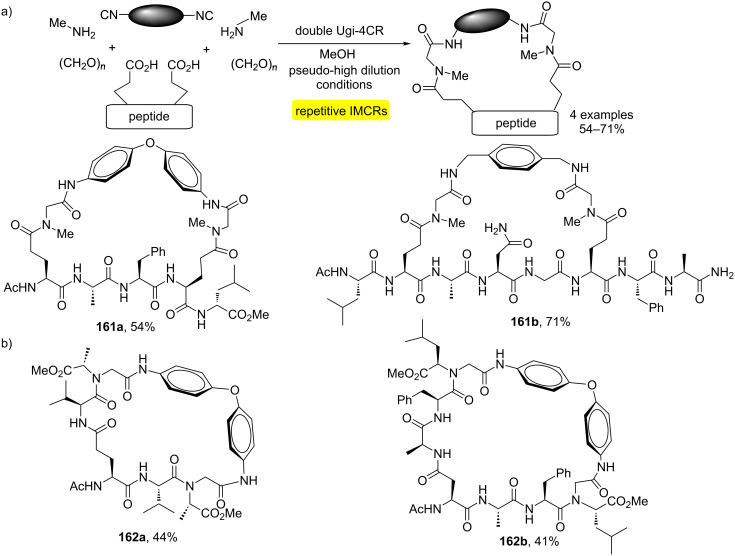
a) Bidirectional macrocyclization of peptides by double Ugi reaction. b) Ugi-4CR for the generation of exocyclic diversity during the bidirectional macrocyclization [48].

Another method using repetitive Ugi reactions has been described for the macrocyclization of peptides [48]. The approach was based on double Ugi-4CR involving a peptide diacid, a diisocyanide, methylamine and paraformaldehyde (Scheme 31a). Subsequently, it was observed that varying the amine component (*C*-protected amino acids) allowed the obtention of exocyclic elements of diversity as observed in macrocycles **162a** and **b** (Scheme 31b). The process allows the increase of the peptide sequence as well as the inclusion of conformational constraints.

**Scheme 32:**
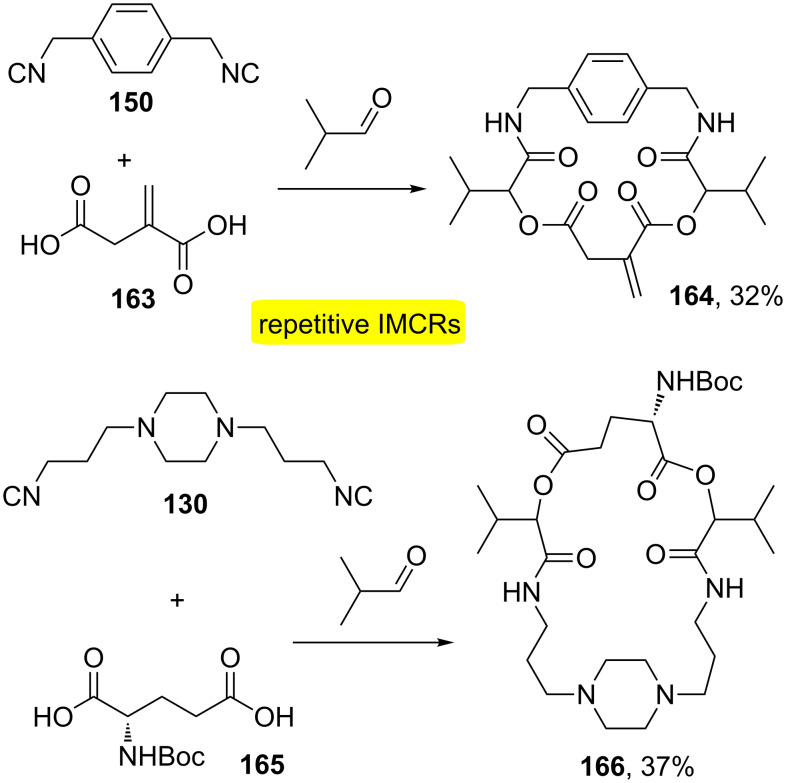
MiBs based on the Passerini-3CR for the synthesis of macrolactones [49].

Wessjohann and co-workers also demonstrated MiBs based on the Passerini-3CR [49]. The strategy is similar to Ugi-MiBs in which it provides molecular diversity in a few steps due to variable combinations of bifunctional building blocks that can be easily changed to provide skeletal diversity in macrocycles. In this approach, two different bifunctional building blocks were combined: diacid/diisocyanide (Scheme 32). This approach also allows the combination of diacid/dialdehyde and dialdehyde/diisocyanide. Macrolactones **164** and **166** were readily obtained using readily available starting materials. Diisocyanides were prepared from commercial diamines in two steps: formylation followed by dehydration of the diformamides. The easiness to obtain the starting materials provides a variety of macrocycles using the Passerini-MiBs.

**Scheme 33:**
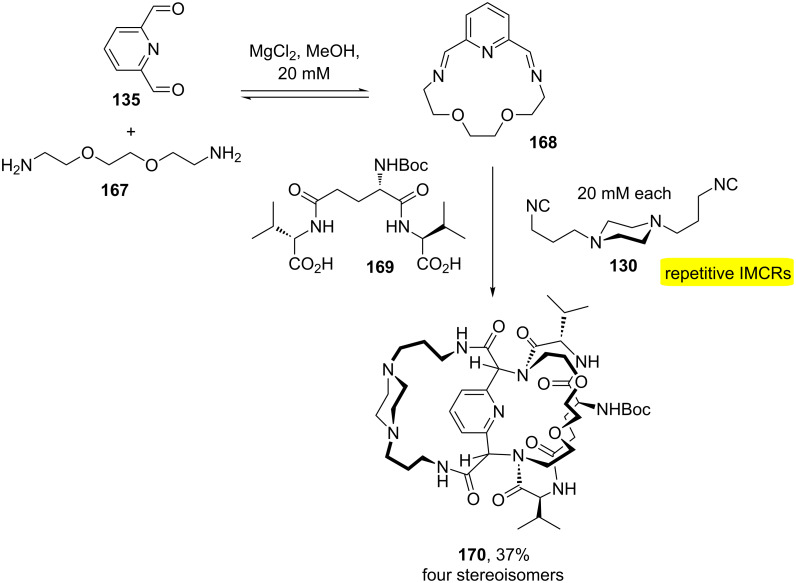
Template-driven approach for the synthesis of macrotricycles **170** [50].

Recently, the use of a double Ugi four-component macrocyclization for the synthesis of molecular cages was described [50]. The approach was based on macromulticycle connectivities through bridgeheads. For the macrocyclization reaction, metal-template-driven and dilution conditions were used. These conditions allowed one-pot synthesis including aryl, heterocyclic, polyether, peptidic and steroidal tethers. Scheme 33 shows a template-driven approach to macrotricycles **170**, which were obtained from preformed diimine **168** and addition of diacid **169** and diisocyanide **130** as building blocks.

## Conclusion

Based on the many examples shown herein, one can conclude that the strategies of repetitive and consecutive isocyanide-based multicomponent reactions can furnish a great variety of different (macro)heterocycles in a short number of steps and with good overall yields in most cases. The strategies are even more powerful when coupled with microwave-mediated reactions, allowing a fast and reproducible synthesis of more complex products. It can be foreseen that this useful strategy will continue to be applied to the synthesis of molecules with even more structural diversity.
